# Deep subsurface microbiology: a guide to the research topic papers

**DOI:** 10.3389/fmicb.2013.00122

**Published:** 2013-05-16

**Authors:** Andreas Teske, Jennifer F. Biddle, Virginia P. Edgcomb, Axel Schippers

**Affiliations:** ^1^Department of Marine Sciences, University of North Carolina at Chapel HillChapel Hill, NC, USA; ^2^School of Marine Science and Policy, College of Earth, Ocean, and the Environment, University of DelawareLewes, DE, USA; ^3^220 McLean Laboratory, Department of Geology and Geophysics, Woods Hole Oceanographic InstitutionWoods Hole, MA, USA; ^4^Department of Resource Geochemistry, Federal Institute for Geosciences and Natural Resources (BGR)Hannover, Germany

Deep subsurface microbiology is a rising field in geomicrobiology, environmental microbiology and microbial ecology that focuses on the molecular detection and quantification, cultivation, biogeographic examination, and distribution of bacteria, archaea, and eukarya that permeate the subsurface biosphere. The deep biosphere includes a variety of subsurface habitats, such as terrestrial deep aquifer systems or mines, deeply buried hydrocarbon reservoirs, marine sediments and the basaltic ocean crust. The deep subsurface biosphere abounds with uncultured, only recently discovered and—at best—incompletely understood microbial populations. So far, microbial cells and DNA remain detectable at sediment depths of more than 1 km and life appears limited mostly by heat in the deep subsurface. Severe energy limitation, either as electron acceptor or donor shortage, and scarcity of microbially degradable organic carbon sources are among the evolutionary pressures that may shape the genomic and physiological repertoire of the deep subsurface biosphere. Its biogeochemical importance in long-term carbon sequestration, subsurface elemental cycling and crustal aging, is a major focus of current research at the interface of microbiology, geochemistry, and biosphere/geosphere evolution. The papers of this Frontiers e-volume bear evidence of the rapid advances in deep subsurface microbiology.

The reader will notice several conspicuous recurring themes in this Frontiers Research topic. This volume opens with a thoroughly argued perspective article on Acetogenesis in the subsurface by Lever (2012), and an accompanying commentary by Oren (2012). Both papers are providing a fresh perspective and a “call to arms” to consider and to investigate acetogenesis as an energetically feasible and widespread pathway that sustains deep subsurface life. These papers argue convincingly that in order to understand the deep subsurface biosphere, major microbial processes in addition to sulfate reduction, methanogenesis and anaerobic methane oxidation have to be explored.

Meanwhile, the microbial cycling of methane and sulfate in the deep subsurface is studied not only with qualitative functional gene surveys but also with functional gene-based molecular quantifications. Schippers et al. (2012) and Blazejak and Schippers (2011) add functional gene qPCR and sequencing to a wide molecular arsenal to quantify and identify the sulfate-reducing and methanogenic microbial communities in organic-rich, reducing marine sediments of Namibia, the Black Sea, and the Peru Margin; the former paper includes an excellent overview on cell counts, qPCR and CARD-FISH quantifications of bacterial and archaeal communities in deep subsurface sediments. In a high-throughput pyrosequencing survey, Mills et al. (2012b) examine the active bacterial community of the methane/sulfate interface in heterotrophic deep marine sediment in the Nankai Trough offshore Japan; by reverse transcription and sequencing of 16S rRNA, they show that the simultaneous presence of methane and sulfate impacts the active bacterial subsurface community only minimally—obviously, other geochemical parameters need to be accounted for. In contrast, hydrocarbon seeps are the extreme endmember of a reducing sedimentary regime that is indeed dominated by microbial sulfate- and methane cycling. Its world-wide distribution remains to be accounted for fully, and is explored here by Siegert et al. (2011) with an Indonesian example. In a combined cultivation-based and functional gene expression study, Ünal et al. (2012) investigated the trace metal requirements and dosage effects on the activity and diversity of methanogen populations in a terrestrial subsurface coal bed. Fichtel et al. (2012) identify chemolithotrophic sulfate-reducing bacteria from deep sediments at the Juan de Fuca Ridge flanks by enrichment, pure culture isolation and physiological testing in the laboratory.

Many microbial groups in the subsurface have no apparent connection to sulfate reduction and methane cycling, but they occur nevertheless in considerable abundance and ubiquity. Two studies of the coastal subsurface—a comprehensive examination of microbial community structure by qPCR, cloning and sequencing of functional and 16S rRNA genes in terrestrial deep sediments near Chesapeake Bay by Breuker et al. (2011), and a reverse transcript analysis of archaea in the methane/sulfate interface of estuarine sediments in Southern China by Li et al. (2012)—focused much of the overall effort on rRNA-based diversity analyses of the Miscellaneous Crenarcheotal Group (MCG), perhaps the most widespread archaeal group in subsurface sediments. The MCG archaea are at present the focus of single-cell sequencing analyses and metagenomic surveys, with the goal of inferring their metabolisms and culture requirements.

Most microbial surveys have focused on organic-rich subsurface sediments in nearshore or continental margin locations where anaerobic processes and populations could be more easily detected. At the opposite end of the spectrum, the oligotrophic subsurface—the organic-lean, non-sulfidic or oxidized sediments of the open ocean which are representative for most of the seafloor and its sedimentary subsurface—is increasingly scrutinized by microbial community structure analyses, as performed in a pyrosequencing survey by Hoshino et al. (2011) in sediments of the Porcupine bight of the Eastern North Atlantic, and with heterotrophic activity measurements in central North-Atlantic deep sea sediments by Picard and Ferdelman (2011). The oligotrophic subsurface constitutes indeed a biogeochemically and microbiologically distinct habitat: in a theory and hypothesis article, Durbin and Teske (2012) synthesize the evidence for a phylum-level archaeal community shift from organic-rich and reduced sediments to organic-lean and oxidized marine subsurface sediments, and provide detailed phylogenies on many seldom-seen archaeal lineages from organic-lean sediments and hydrothermal vents.

Deep subsurface microbiology does not stop at the sediment/bedrock interface. The basaltic ocean crust is permeated by cracks and fissures that provide microbial habitat and an interface where rock-hosted microbial communities chemically modify the rock matrix. Viewed over long time frames, microbes act as a geological force that catalyzes the chemical alteration of the upper ocean crust, as summarized by Edwards et al. (2012). The energy reservoir of the deep basalt biosphere is demonstrated by a bore hole observatory at Hole ODP896A in the basaltic crust of the Costa Rica Rise; its microbial ecosystem containing a large proportion of autotrophic sulfur oxidizers is sustained by subsurface mixing of reduced formation fluid and oxic seawater—a discovery by Nigro et al. (2012). In a comprehensive metagenomic survey of a different type of rock-hosted microbial ecosystem, Brazelton et al. (2012) are exploring the methanogenic and hydrogenotrophic microbiota fuelled by serpentinization reactions, focusing on terrestrial serpentinite springs on Newfoundland and comparing their metagenomes to those of the previously studied Lost City vents. The rock-hosted terrestrial subsurface biosphere is often more accessible than its marine counterpart, and allows repeated biogeochemical and microbial sampling in mines and boreholes; such accessibility facilitates targeted investigations of specific pathways and processes, for example nitrogen fixation and nitrification in deep granite-hosted ores in Colorado studied by Swanner and Templeton (2011). An overlooked extreme subsurface habitat that requires more microbiological study are mile-deep ice cores; their microbial inhabitants are introduced here by an Ice-binding protein study of a *Flavobacterium* isolated from the deepest ice core segments (close to rock basement) at Station Vostok in Antarctica by Achberger et al. (2011).

Several papers in this volume are addressing methodological advances in deep subsurface microbiology, which are crucial for further progress in this field. New hardware for high-pressure microbial incubations, plus instructions how to build this in your own workshop, is being introduced by Sauer et al. (2012); caution in the long-term storage of microbial samples at 4°C is advocated by Mills et al. (2012a) after they documented storage-related microbial community shifts by high-throughout sequencing; and previously published cell counting protocols for deep subsurface sediments are further refined by Lappé and Kallmeyer (2011) specifically for hydrocarbon-rich sediments.

The volume concludes with papers that provide an evolutionary and genomic perspective on the deep microbial biosphere. Biddle et al. (2012) ponder the future of microbial evolution studies in the subsurface; they advocate to select and study model organisms and phylogenetically defined groups within defined environmental gradients as a promising strategy. To facilitate big-picture genomic surveys, Martino et al. (2012) introduce a novel degenerate PCR primer strategy for metagenomic recovery of unknown microbial communities from the deep subsurface. Anderson et al. (2011) integrate and review the evidence for viral infection of subsurface bacteria and archaea, and for a substantial viral contribution to hydrothermal vent and subsurface metagenomes, while reflecting on the different selection pressures that shape viral communities in these habitats.

This e-volume includes both new methodologies and research strategies that should yield promising results in the future, and expands our current view of deep subsurface microbiology with innovative studies of a wide range of subsurface habitats and microbial ecosystems. There is ample room for further exploration; we note that several areas in subsurface microbiology remain severely understudied. The eukaryotic, mostly fungal subsurface biosphere has only recently been discovered and is being investigated by volume editors Edgcomb and Biddle. The exploration of the deep biosphere in the ocean crust has just started and opens major research opportunities. Unlike marine sediments where photosynthesis-derived buried organic carbon sustains microbial life, inorganic processes in the ocean crusts likely provide the main energy sources for the rock-hosted deep biosphere. The terrestrial deep subsurface and the oceanic crust biosphere remain to be quantified, and overall microbial diversity and function of these microbiota remain to be accounted for across a wide range of geological settings and sampling sites. Everywhere in the subsurface, the study of microbial physiology, the cultivation of novel types of microorganisms, and biochemical analyses of subsurface life strategies remain high on the list of current and future research challenges. Overall, continued in-depth exploration of subsurface biomes will continually yield new insights on microbial adaptation and survival under extreme conditions.
